# P04.34. Should Medicare expand allowable chiropractic services?

**DOI:** 10.1186/1472-6882-12-S1-P304

**Published:** 2012-06-12

**Authors:** J Whedon

**Affiliations:** 1 Dartmouth College, Lebanon, USA

## Purpose

Chiropractic care is covered under Medicare, but the scope of services is restricted. The Demonstration of Expanded Coverage of Chiropractic Services under Medicare projected that expanded services would cost Medicare up to $1.15 billion annually. The potential expansion of chiropractic services under Medicare remains controversial. Advocates for expansion cite clinical effectiveness and high patient satisfaction, while critics point to compliance issues and projected cost increases.

## Methods

Seven reports from the US Department of Health and Human Services and the results of two focused Medline queries were reviewed for evidence related to the provision of chiropractic care under Medicare. The literature review summarized the findings of government reports and peer-reviewed publications, and explored the pros and cons of expanded services.

## Results

Three problem areas pose barriers to expansion of chiropractic services under Medicare: (1) putative provision of maintenance (wellness) care in apparent violation of Medicare practice guidelines; (2) inadequate clinical documentation; and (3) projected increased costs associated with expanded services. However, maintenance care is poorly defined, and evidence for its clinical effectiveness is not without promise, although its cost-effectiveness is unknown. Inadequate clinical documentation, although not justifiable, may be connected to disparities in Medicare documentation requirements and reimbursement. The analysis of budget neutrality of the demonstration project may have been subject to bias due to selection of unrepresentative demonstration sites. No causal relationship was established between expanded services under the demonstration and the observed differences in total Medicare costs.

## Conclusion

Research on costs and clinical outcomes associated with maintenance care is needed. Any unjustifiable disparities in coverage and payments under Medicare should be corrected. Chiropractic physicians must assume responsibility for correcting deficiencies in compliance and documentation. A policy decision on the expansion of chiropractic services under Medicare should consider the results of a re-evaluation of the demonstration, which is currently underway.

